# Diet-Induced Cognitive Deficits: The Role of Fat and Sugar, Potential Mechanisms and Nutritional Interventions

**DOI:** 10.3390/nu7085307

**Published:** 2015-08-12

**Authors:** Jessica E. Beilharz, Jayanthi Maniam, Margaret J. Morris

**Affiliations:** Department of Pharmacology, School of Medical Sciences, UNSW Australia, UNSW Sydney 2052, Australia; E-Mails: j.beilharz@unsw.edu.au (J.E.B.); j.maniam@unsw.edu.au (J.M.)

**Keywords:** diet, memory, hippocampus, fat, sugar, inflammation, neurogenesis, BDNF, intervention, omega-3, curcumin

## Abstract

It is of vital importance to understand how the foods which are making us fat also act to impair cognition. In this review, we compare the effects of acute and chronic exposure to high-energy diets on cognition and examine the relative contributions of fat (saturated and polyunsaturated) and sugar to these deficits. Hippocampal-dependent memory appears to be particularly vulnerable to the effects of high-energy diets and these deficits can occur rapidly and prior to weight gain. More chronic diet exposure seems necessary however to impair other sorts of memory. Many potential mechanisms have been proposed to underlie diet-induced cognitive decline and we will focus on inflammation and the neurotrophic factor, brain-derived neurotrophic factor (BDNF). Finally, given supplementation of diets with omega-3 and curcumin has been shown to have positive effects on cognitive function in healthy ageing humans and in disease states, we will discuss how these nutritional interventions may attenuate diet-induced cognitive decline. We hope this approach will provide important insights into the causes of diet-induced cognitive deficits, and inform the development of novel therapeutics to prevent or ameliorate such memory impairments.

## 1. Introduction

Obesity is one of the most serious and costly health challenges facing the modern world. Energy intake has increased in recent times through the high availability and low cost of energy dense, nutrient poor foods and drinks [1,2]. On the other side of the scale, energy expenditure has decreased through changes in urban design and an increased reliance on labour saving devices such as cars [3]. With this obesogenic environment in mind, it is alarming but almost unsurprising that over 1 billion adults worldwide are overweight [4]. Obesity costs the global economy around $2 trillion annually, which is equivalent to 2.8% of worldwide gross domestic product [5].

Obesity is an important determinant of a range of health disorders. Obesity is clinically defined based on measurements of body mass index (BMI, overweight ≥25; obese ≥30) [6]. However, it can more broadly be described as the physiological condition in which body fat has accumulated to an extent that is detrimental to overall health [7]. Being overweight increases the risk of metabolic syndrome, type 2 diabetes, cardiovascular disease, some cancers, respiratory conditions, fatty liver disease, reproductive disorders, depression and other mental health conditions. Recent evidence has also highlighted that obesity is associated with cognitive impairments and an increased risk of developing dementia and Alzheimer’s disease later in life. These topics have been reviewed in detail elsewhere [8,9].

The current review will discuss the issue of whether or not obesity is necessary for these cognitive deficits and, if obesity is not necessary, what other variables are mediating this effect? As the brain cannot synthesise or store energy reserves, food provides its immediate source of energy and thereby may influence structure and function [10]. Several interactive processes have been proposed to underlie cognitive decline related to poor diet including oxidative stress and inflammation [11], increased blood brain barrier permeability [12,13], reduced neurotrophic factors [14], and insulin insensitivity [15,16]. For the purpose of this review, we will focus on inflammation and neurotrophic factors.

Finally, while a range of nutritional interventions have shown beneficial effects on memory status in human and animal models, here we will focus on omega-3 and curcumin, specifically discussing the mechanisms underlying the positive effects of these nutrients on memory and highlighting potential future work.

## 2. Is Obesity Necessary for Memory Deficits?

There has been considerable interest of late in the relationship between obesity and cognition. The PubMed database highlights 1442 items from a search of the terms “obesity” and “cognition” and when this is narrowed to the past 10 years, 1127 items remain. These articles generally conclude that obesity is associated with worse cognitive performance however the exact mechanisms behind this relationship remain unknown.

In middle age, obesity is generally associated with cognitive impairments independently of other socioeconomic or health related factors. For instance, Cournot, *et al.* [17] showed that higher BMI was associated with lower cognition scores on word-list learning and the Digit-Symbol Substitution test in healthy, middle-aged, non-demented subjects (aged 32, 42, 52, 62 years). At 5-year follow-up, higher baseline BMI was associated with greater cognitive decline on word-list learning. Similarly, Dahl, *et al.* [18] using the Swedish Twin Registry, showed that a higher BMI in middle-age (mean age 41) predicted lower performance and steeper decline 18 years later on several cognitive domains including long and short term memory, speed, verbal and spatial ability. Similarly, in an older age group (50–60 years) from the same registry, mid-life BMI predicted cognitive performance 30 years later however, baseline BMI was only associated with steeper decline on long term memory [19]. Earlier time-points were not examined in this registry, which raises the question of when the relationship between obesity and cognition manifests. These studies tentatively suggest that mid-life BMI may be more important for predicting cognitive decline than BMI later in life.

Cross-sectional studies on older age cohorts show more inconsistent results. Some studies tentatively indicate that higher BMI is associated with better cognition [20,21]. For example, in an American community based study sampling people ≥65 years, higher BMI was associated with less cognitive decline on the Mini-Mental State Examination (MMSE) at 6 years follow up. However, when the analyses were adjusted for comorbid illnesses, these associations were much smaller and were not significant in participants with normal cognition at baseline [22]. Other studies have shown that higher BMI is associated with poorer cognition independently of variables such as hypertension, diabetes and medications that may affect cognition. For example, in a Spanish community sample aged ≥65 years, higher BMI was associated with worse performance on the MMSE and other cognition measures [23]. However, other work has shown that the relationship between obesity and cognition is attenuated or better explained by facets of metabolic syndrome including inflammation [24] or cardiovascular disease including hypertension [25,26].

While obesity may be a risk factor for cognitive decline, others factors may mediate this effect. As previously discussed, obesity is associated with an increased risk for a number of conditions including metabolic syndrome, systemic inflammation and reduced cardiovascular fitness. These conditions are under-diagnosed in the general population and issues arise when they are not identified or controlled for in studies because, even in healthy populations, they have the potential to negatively impact cognition [27,28,29] and thus skew results. Moreover, many older participants are diagnosed with dementia or may be in the pre-clinical phase, which is often undiagnosed and accompanied by weight loss [30]. We believe that it would be beneficial for these studies to consider factors which may lead to obesity including dietary intake, rather than simply diseases that are comorbid with obesity.

## 3. Does the Food We Eat Impair Our Cognition?

The current obesity epidemic is characterised by super-sized, cheap, extremely palatable, high-energy foods. People are often ill informed regarding nutritional labelling and the recommended dietary guidelines, and can easily incorrectly believe they are making healthier choices by clever marketing strategies such as “low fat” products. In this context, it is important to examine the ways in which both total energy intake and the macronutrient profile of the food may be influencing cognition.

Of concern is that a Western style diet high in saturated fat and refined sugars can impair certain types of memory in healthy subjects. Francis and Stevenson [31] for example showed that healthy undergraduate student participants with high self-reported fat and refined sugar intake were impaired on memory tasks that were sensitive to hippocampal function. These tasks included the verbal paired associates and logical memory subsets of the Wechsler Memory Scale Revised. In addition, in a laboratory-based test of food intake, they were less accurate at recalling what they had previously eaten and had reduced sensitivity to internal signals of hunger and satiety, as they had to eat more before they reported the same level of satiety. These tasks are also hippocampal-dependent because the hippocampus has a role in episodic memory (*i.e.*, remembering what we have eaten) and our responsiveness to internal hunger and satiety cues.

Similar results have been found in school-aged children. In an Australian cohort, higher intake of a Western style diet at age 14 was correlated with worse cognitive performance 3 years later. These associations were found on tasks that assessed visual spatial learning, long-term memory and reaction times. Moreover, these associations remained significant when adjusted for possible confounding variables including BMI and exercise level [32]. Others have found comparable results in cross-sectional designs. For example, Overby, *et al.* [33] reported that students with higher intakes of a Western diet had more self-reported difficulties in mathematics.

If we now focus on the macronutrient profile of the food, higher intakes of saturated fatty acids (SFA) have also been associated with cognitive impairment. Cross-sectional and longitudinal correlational studies indicate that higher intakes of SFA in young adulthood, mid and later life are associated with worse global cognitive function, impairments in prospective memory, memory speed and flexibility and an increased vulnerability to age related deficits and neurological diseases including dementia and Alzheimer’s disease [34,35,36,37]. In contrast, higher intakes of polyunsaturated fatty acids (PUFA) and higher PUFA to SFA ratios are associated with better memory function (even in children) and a reduced risk of memory impairment [34,38,39,40]. More specifically, higher intakes of omega-3 PUFA compared to omega-6 PUFA have been linked to a lower risk of cognitive decline and Alzheimer’s disease [41,42,43]. These studies indicate that the quality and type of fat may impact cognition more than total fat intake but this is difficult to ascertain in human populations.

Likewise, higher intakes of carbohydrates, particularly simple sugars, have been associated with lower cognitive function. In a population-based prospective cohort of elderly people, the risk of mild cognitive impairment or dementia was increased in subjects who derived a high percentage of their energy from carbohydrates but was reduced in those who derived a high percentage of energy from fat and protein [44]. Similarly, a cross-sectional study on school children (aged 6–7 years) reported an inverse relationship between the consumption of refined carbohydrates and non-verbal intelligence after adjustment for factors such as BMI. These tests measured skills such as the children’s ability to organise spatial perceptions and how they stored and reproduced information [45].

In humans, only a limited number of experiments have controlled participant’s diets and assessed cognition. Two studies have investigated the acute effects of a high fat diet (approximately 75% of energy) on memory, attention and mood. In healthy, young adult male subjects this diet was sufficient to impair attention, speed of retrieval and depress mood after only 5 days [46]. Similarly, in sedentary male subjects (<2 h/week physical activity), 7 days of the same diet decreased reaction times and attention [47]. In regards to sugar, Wolraich, *et al.* [48] found that 3 weeks of a diet sweetened with sucrose (5600 mg/kg/day), saccharin (12 mg/kg/day) or aspartame (38 mg/kg/day) had no behavioural or cognitive effects on children. However, other studies have found that a single meal with a high glycaemic load can impair memory performance in children and healthy young adults [49,50].

Together these studies highlight that excessive energy can impair cognition but importantly, diets high in either fat or sugar can also impair cognition. The experimental studies in humans also show that these memory deficits can occur rapidly, within 1 week of diet exposure, and therefore arise independently of any effects on body weight or general health.

## 4. How Rapidly Do Diet-Induced Cognitive Deficits Occur?

The difficulties in separating the effects of diet from other sociocultural influences in human studies means that animal models are necessary to further elucidate how rapidly the effects of diet on memory can emerge. Animal studies not only allow strict control over diet and other external factors but a range of memory tasks are available to assess different brain areas such as the hippocampus and molecular changes within the relevant regions can be examined. Nevertheless, to date, comparisons between animal studies have been difficult due to the variations in the fat and/or sugar source and concentrations, the length of diet, the behavioural tests used and the rodent species and age. Moreover, only a handful of studies have directly compared different sorts of high-energy diets within the same experiment or have assessed the effect of diet length on different types of memory. This section of the review will focus on studies that have addressed these questions in adult rats.

Rodent studies indicate that the hippocampus may be particularly vulnerable to the effects of high-energy diets. Kanoski and Davidson [51] showed rapid and stable spatial impairments in rats performance on the radial arm maze (RAM) from 3 to 90 days on a high fat and glucose diet. In contrast, over 30 days exposure to the diet was necessary for stable impairments on the non-spatial version of the task. Likewise, we have shown impairments on the hippocampal-dependent place recognition task within 5 days of commencing a cafeteria diet (with or without 10% liquid sucrose), or a chow diet supplemented with liquid sucrose. These groups however were able to identify a novel object on the perirhinal-dependent object recognition task after 5, 11 and 20 days of diet. After 1 month of diet, there were no diet effects on hippocampal expression of neurotrophic markers such as brain derived neurotrophic factor (BDNF) but there were large inflammatory changes. Specifically, our cafeteria plus sucrose fed rats had elevated hippocampal tumor necrosis factor alpha (TNF-α) and interleukin-1 beta (IL-1β) mRNA compared to control rats, this tended to be higher in the sucrose only rats but no effect was seen in the cafeteria only rats. These inflammatory changes were strongly correlated with place recognition deficits [11].

Only a handful of studies have directly compared the effects of fat and sugar on memory. Jurdak, *et al.* [52] assessed spatial and cued performance in the Morris Water Maze (MWM) after 5 weeks exposure to high sucrose or high fat chow. On the spatial version of the task, the sucrose and fat groups had longer escape latencies than controls on a retention test 1 day after training, and the sucrose group was also impaired 3, 4 and 10 days after initial training. Both groups performed in line with controls on the non-spatial cued task. Moreover, using the same diets in a separate study they found deficits on the perirhinal-dependent object recognition task after 8 weeks on the sucrose but not fat supplemented diet [53]. These studies respectively reported a positive correlation between glucose tolerance and escape latencies on the MWM [52] and a negative correlation between fasting blood glucose and the amount of time exploring a novel relative to familiar object [53]. Sugar may therefore impair hippocampal-dependent and independent memory prior to changes in response to a high fat diet via its more rapid alterations in insulin sensitivity and glucose metabolism.

Some recent studies have also directly compared the effects of different sorts of sugars. Kendig, *et al.* [54] examined the acute effects of isocaloric solutions of sucrose (10%) or maltodextrin (10.4%) on object and place recognition memory. Maltodextrin, like sucrose, is a rapidly absorbed carbohydrate but it does not contain fructose. The rats in both sugar conditions were impaired on the place, but not object task, after 17 days on the diets. These results suggest that fructose is not necessary for diet-induced cognitive deficits. Unfortunately no molecular analyses were performed on these rats. In contrast, Hsu, *et al.* [55] showed that a standard diet supplemented with high fructose corn syrup (HFCS, 11%) was more harmful to memory than a diet supplemented with liquid sucrose (11%). Whilst both groups were impaired on the hippocampal-dependent Barnes maze, the HFCS group showed longer escape latencies on 3 trials and the probe test while the sucrose group was only impaired on 1 trial. Both groups performed in line with controls on the non-spatial Y-maze and showed similar anxiety levels on the zero maze. These deficits were related to elevated hippocampal inflammation (IL-1β and interleukin-6 (IL-6) protein) only in the HFCS rats. Importantly, the memory deficits and inflammation were only evident in adolescent, not adult rats, indicating that this period of development may represent a sensitive window.

We therefore propose that obesity does not influence cognition directly, rather, consuming a diet high in fat and/or sugar initiates a number of more rapidly occurring central and peripheral changes such as inflammation which mediate this effect. The hippocampus is particularly vulnerable given its crucial role in memory, and other factors may include its location and lack of vascularisation [56]. Possible factors driving these impacts are discussed below.

## 5. What Mechanisms May Be Responsible for Diet-Induced Cognitive Deficits?

### 5.1. Inflammation

Memory loss is not solely induced by the accumulation of specific risk factors but rather, it is a manifestation of a gap between increased demand due to cumulative insult, risk factors or injury, and the inability of the immune system to meet these increased needs [57]. Microglia act as sensors within the brain to detect deviations from normal homeostasis and they are among the first cells to respond to pathological events. Microglia become transiently activated when a deviation is detected which allows them to migrate to the site of insult and initiate a number of immune functions including the production of both cytotoxic and neurotrophic factors [58]. Importantly, this activation can induce a priming or sensitising effect in which subsequent insults results in greater microglia activation [59,60].

Obesity and its comorbidities, including type 2 diabetes mellitus and cardiovascular disease, are characterised by a state of chronic low-grade inflammation [61,62,63,64]. Interestingly, individuals with metabolic syndrome that show increased inflammation have more pronounced cognitive deficits than those with low levels of inflammation [24]. Moreover, several population-based epidemiological studies have shown that high levels of cytokines can actually pre-date and anticipate the development of over-nutrition related diseases such as diabetes [65]. Inflammation may therefore actually hold a predictive value, rather than simply being a consequence of obesity.

In line with this, experimentally induced inflammation can cause memory deficits. Reichenberg, *et al.* [66] for example, showed impaired global memory in healthy subjects injected with a bacterial endotoxin and their performance was positively correlated with cytokine secretion. Similarly, in rodent studies, pharmacologically induced inflammation impairs memory however, these deficits are generally specific to hippocampal-dependent tasks. For example, Gibertini and colleagues showed that peripheral injections of IL-1β are sufficient to impair spatial but not non-spatial MWM performance. Moreover, while the saline injected mice flexibly adjusted their behaviour when the platform position was moved, IL-1β injected mice continued to look for the platform in its previous position. Perseverance errors are another measure of hippocampal-dependent memory [67,68]. Likewise, intra-cerebral administration of IL-1β has been shown to impair contextual (hippocampal-dependent) but not auditory-cued (hippocampal-independent) fear conditioning [69,70,71]. These rodent studies suggest that the hippocampus is particularly sensitive to inflammation and are supported by other studies which show that the hippocampus has a particularly high level of cytokine binding [72].

Moreover, high fat and sugar diets can rapidly induce inflammatory changes in rodent models. Thaler, *et al.* [73] showed that 1 to 3 days of high fat feeding was sufficient to induce microglia activation and change mRNA expression of inflammatory markers in the hypothalamus. This acute response temporarily subsided around 2 weeks but with continued feeding, it was re-established giving way to chronic inflammation, neuron loss and reactive gliosis. Another study examined the hippocampus of rats fed high fat pellets (10% lard) plus a 20% high-fructose corn syrup solution for 1 week. In the hippocampus they found significant increases in reactive astrocyte number and size but only slight, non-significant changes in microglia cells [16]. We have also shown hippocampal inflammation after 1 month of a cafeteria diet plus 10% liquid sucrose or a chow diet supplemented with liquid sucrose. These changes were strongly correlated with memory deficits on the hippocampal-dependent place task [11]. Similarly, Hsu, *et al.* [55] reported larger hippocampal-dependent memory deficits after 1 month of a control diet supplemented with HFCS, than with sucrose, and these deficits were related to hippocampal inflammation in the HFCS, but not sucrose, rats. Chronic high-energy diet studies have also shown a relationship between hippocampal inflammation and memory deficits [74,75].

### 5.2. Neurotrophic Factors

BDNF plays an important role in the survival, maintenance and growth of many types of neurons and is expressed abundantly in the hippocampus, hypothalamus, and cerebral cortex [76]. BDNF affects neuronal plasticity through molecules such as synapsin 1, growth associated protein 43 (GAP-43) and cAMP response element-binding protein (CREB). Through its phosphorylation of synapsin 1 for example, BDNF facilitates neurotransmitter release, axonal growth and the formation and maintenance of presynaptic structures [77,78]. BDNF also plays a role in gene expression and long term memory through its phosphorylation of CREB [79].

Diets high in fat and sugar reduce BDNF expression and this has been correlated with memory deficits. Molteni, *et al.* [14] for example, found reduced hippocampal, but not cerebral cortex, BDNF mRNA and protein after 2 months, 6 months and 2 years on a high fat and sucrose diet in rats. Downstream effectors including hippocampal synapsin 1, GAP-43 and CREB mRNA were also reduced proportionally to BDNF. The lowest levels of these markers were recorded at 2 years. Longer escape latencies on the MWM were correlated with lower levels of BDNF mRNA and protein in the hippocampus.

Nevertheless, it is not clear how a high-energy diet translates into decreased BDNF levels. Molteni, *et al.* [10], using the same protocol as their previous study [14], demonstrated that diet-induced decreases in BDNF, synapsin 1, GAP-43, CREB and spatial cognition were reversed in rats allowed voluntary running wheel access during the 2 month diet period. In addition, they found lower levels of oxidative stress in rats with access to the high-energy diet plus the running wheel. They therefore proposed that oxidative stress was one of the earliest events following the consumption of a high-energy diet and that reactive oxygen species formation may play a role in BDNF reduction and cognitive dysfunction.

Other studies support the claim that diet-induced changes in BDNF expression may be mediated by inflammatory cytokines. Hippocampal BDNF expression following contextual learning for instance is blocked by intra-hippocampal IL-1β administration [69]. The literature also suggests that while cytokines can be rapidly induced by diet, more chronic diet exposure is necessary before decreases are seen in neurotrophic factors. For example, we have seen large inflammatory, but not neurotrophic changes (e.g., BDNF) after 1 month of diet [11]. Other studies have reported decreased BDNF, CREB, GAP-43 and synapsin 1 mRNA and protein after 2 months of a high fat and sugar diet [14].

## 6. Interventions to Prevent or Reverse Diet-Induced Memory Deficits

The beneficial contribution of diet to mental health and cognition has long been recognised and is well studied [42,80]. Recently dietary interventions, particularly long-chain polyunsaturated fatty acids (LCPUFA) and curcumin, have been targeted at preventing and reversing the cognitive deficits seen with ageing. These dietary interventions have been widely shown to reduce inflammation and/or improve neurogenesis [81,82,83]. Given the strong links between increased inflammation and reduced neurogenesis with diet-induced cognitive deficits [10,11], the efficacy of LCPUFA and curcumin on preserving or preventing cognitive deficits will be assessed. Less work has explored the effects of these properties on diet-induced memory impairments.

### 6.1. Long-Chain Polyunsaturated Fatty Acids

In humans, LCPUFA are important for maintaining mental health as they play a critical role in brain development, particularly in the integrity and function of the neuronal membrane [84]. Docosahexaenoic acid (DHA; 22:6 omega-3) and arachidonic acid (ARA; 20:4 omega-6) are the most abundant LCPUFA in the brain [85].

A low dietary ratio of omega-3/omega-6 has been associated with an increased risk of cognitive impairments [41,86] and dementia [87,88]. For example, in a free living subpopulation aged between 63–74 years, omega-3/omega-6 plasma ratio was inversely correlated with cognitive decline during a 4 year follow up [41]. In cross-sectional studies, lower plasma omega-3/omega-6 was observed in patients who were suffering from dementia compared to non-demented patients [89,90]. It is noteworthy that normal ageing is accompanied by a reduction in DHA levels in the brain and a decline in memory [41,91]. Hence, there is a strong association between omega-3/omega-6 ratio with cognition in healthy and disease related conditions.

Emerging data from clinical trials has demonstrated that supplementation with omega-3 plays a role in improving cognitive status in young adults, in the maintenance of normal cognitive function and in the prevention of age-related cognition deficits [92,93,94,95,96]. However, supplementation with omega-3 produces differential effects on cognitive function depending on the age, and health status of individuals tested. For example, in healthy elderly individuals, omega-3 supplementation did not improve cognition [97,98] but significant improvements have been observed in people suffering from mild-cognitive deficits [99,100,101]. Other studies have reported no cognitive benefits in patients with Alzheimer’s disease [102,103].

The differential effects of omega-3 supplementation on cognitive function are driven by several factors. One major reason for different observations across studies could be the duration of the supplementation. Work in animal models, most of which reported a cognitive benefit of omega-3, provided supplementation to the animals for more than 10% of the total lifespan of the animal [104]. The type of diet consumed also affects the level of DHA one receives. For example, the western diet that humans commonly consume has high omega-6/omega-3 PUFA ratios. It has been estimated the ratio of omega-6/omega-3 PUFA in western diet is 15-20:1 instead of 1:1 [105]. Foods such as flaxseed oil, nuts and green, leafy vegetables are rich in omega-3 [106] while omega-6 is high in vegetable oil products such as sunflower seed oil, soybean, corn, palm, canola oils, margarine and shortening which are common ingredients of the western diet [107]. Previous work has shown that a high fat diet increases plasma and brain omega-6/omega-3 PUFA ratios [108]. Also, dams exposed to a high omega-6/omega-3 PUFA diet perinatally had offspring with high hippocampal omega-6/omega-3 PUFA ratio and decreased neurogenesis and hippocampal plasticity-related genes such as doublecortin (Dcx) and cluster differentiation 200 (Cd200). In addition, the offspring that also consumed a high fat diet post-weaning had impaired spatial memory [109].

In addition, there is currently a transition in the food industry where sugar is added to many foods including processed meat, sauce, dairy products and fruit juice. While the implications of an unbalanced dietary omega-3/omega-6 PUFA ratio on metabolic deficits is well documented [84,85,86,87,88], the combined effects of an unbalanced omega-3/omega-6 ratio diet plus high sugar consumption is less explored. There is one animal study which showed high sugar (fructose) consumption combined with an omega-3 deficient diet led to memory impairments [110]. In this study, spatial learning and memory retention was assessed by measuring latency time in the Barnes maze, and in those mice fed an omega-3 deficient diet, latency time was significantly increased versus those fed a replete diet, indicative of memory impairment. This memory impairment was worsened by fructose intake [110]. The fructose induced memory impairment was also associated with diet-induced metabolic derangements including elevated insulin and lipid profile [110,111].

As examining the mechanisms underlying the effects of diets comprising high omega-3/omega-6 ratio on cognitive performance is impossible in humans, animal models are necessary. Rodent studies have shown the cognitive benefits of a high ratio of omega-3/omega-6 PUFA supplementation on hippocampal-dependent memory tasks such as spatial recognition [80,81,82]. There are several mechanisms that are likely to regulate improved cognition following DHA supplementation. DHA can cross the blood brain barrier and is highly concentrated in the adult brain. In rodents, for example, it represents about 15%–20% of the total brain lipids [112]. DHA can affect neuronal differentiation by promoting neurite growth in hippocampal neurons [113,114] and hence has the potential to increase neurogenesis. Omega-3 supplementation increases molecular markers involved in plasticity including BDNF and tropomyosin receptor kinase B (TrkB) [80]. Omega-3 supplementation also works through preventing neuroinflammatory processes. There is evidence in mice that dietary omega-3/omega-6 ratio is inversely associated with cognitive decline and hippocampal inflammation [82]. Specifically, after 4 months of supplementation, hippocampal TNF-α positive cells were reduced in a dose dependent manner. Mice consuming a high omega-3/omega-6 ratio diet had fewer hippocampal TNF-α positive cells and this coincided with significantly lower hippocampal TNF-α mRNA expression [82]. In another study, 2 months of an omega-3 enriched diet restored age related spatial memory deficits. This was associated with increased hippocampal DHA levels, and a reduction in hippocampal inflammatory markers such as TNF-α and IL-1β mRNA [81].

### 6.2. Curcumin

Curcumin is a natural polyphenol and a popular Asian dietary spice used in curry and traditional Chinese medicine [115]. Its role in regulating cognition function has only received wide attention in the last 10 years. Most studies examining the impact of curcumin have addressed age-related cognitive decline [83,116] or cognitive deficits induced by an insult either through cigarette smoking [117] or chemicals such as nitric oxide [118], phenobarbitone [119], carbamazepine [119] or stress [120]. Curcumin supplementation has been consistently reported to produce beneficial effects in either ameliorating or rescuing cognitive deficits imposed by the exposures/conditions outlined above. Whether curcumin has a positive effect on diet-induced memory impairments remains to be explored.

Curcumin’s effect on cognitive performance may involve several mechanisms or pathways. For instance, curcumin has been shown to improve cognition via its effect on synaptic plasticity [83,120,121]. Curcumin improves synaptic plasticity through alterations in key proteins such as calcium/calmodulin-dependent kinase II (CaMKII) and *N*-methyl-d-aspartate receptor (NMDAR) [121]. Curcumin can also reduce oxidative stress, which is associated with improved cognition [119,122]. A very recent study demonstrated that curcumin promotes the synthesis of DHA from its precursor, α-linolenic acid (C18:3 n-3; ALA) and increased the activity of enzymes involved in the synthesis of DHA such as fatty acid desaturase 2 (FADS2) and elongase 2 in the brain [123]. The same research group has previously demonstrated beneficial effect of curcumin in preventing reduced brain DHA levels after brain trauma [124].

In summary, both DHA and curcumin have some positive effects on cognitive function in healthy ageing and in disease states, and they appear to target plasticity, inflammation and oxidative metabolism in the brain. Given that diets high in fat and sugar impair memory and are associated with decreased neurogenesis and increased inflammatory responses, DHA and curcumin may represent therapeutic targets in reversing cognitive deficits induced by poor diet.

## 7. Conclusions

Unless the current obesity trend is halted, the burden of chronic disease in future generations will be pandemic and cause a crisis in health and economic systems across the world. It is of vital importance to understand how foods which are making as fat, such as those high in saturated fat and refined sugar, also act to impair cognition and mood (potentially prior to weight gain), and how these deficits can further dysregulate appetite control.

Overeating fat and/or sugar can impair a range of memory functions. In this review we have focused on the hippocampus as a number of studies now highlight that it is particularly sensitive to diet effects. The hippocampus is responsible for a number of functions including episodic memory (*i.e.*, remembering what we have eaten) as well as our responsiveness to internal hunger and satiety cues. Impaired hippocampal function may therefore create a vicious cycle in which it is both a cause and consequence of overeating and obesity. Specifically, diet-induced hippocampal damage can occur prior to weight gain and this may lead to further overeating of the foods that caused the dysfunction in the first place [125,126].

Rapid onset diet-induced hippocampal deficits may be related to inflammation in the central nervous system but further studies are necessary to ascertain this. Further research is also necessary to determine the mechanisms through which obesity and its comorbidities may exacerbate cognitive impairments. In this review, we have shown that omega-3 and curcumin may provide promising targets to attenuate or prevent these deficits as they act on common targets including inflammation and neuroplasticity. Future studies should investigate this. Together, these results will provide important insights into the causes and interdependencies of mild cognitive impairments and dementia syndromes, and inform the development of novel interventions to ameliorate and prevent these disorders.
